# A microsatellite-based linkage map of salt tolerant tilapia (*Oreochromis mossambicus* x *Oreochromis spp*.) and mapping of sex-determining loci

**DOI:** 10.1186/1471-2164-14-58

**Published:** 2013-01-28

**Authors:** Feng Liu, Fei Sun, Jian Li, Jun Hong Xia, Grace Lin, Rong Jian Tu, Gen Hua Yue

**Affiliations:** 1 Molecular Population Genetics Group, Temasek Life Sciences Laboratory, 1 Research Link, National University of Singapore, Singapore, 117604, Republic of Singapore

**Keywords:** Tilapia, Salt tolerance, Breeding, Sex, Growth

## Abstract

**Background:**

Tilapia is the common name for a group of cichlid fishes and is one of the most important aquacultured freshwater food fish. Mozambique tilapia and its hybrids, including red tilapia are main representatives of salt tolerant tilapias. A linkage map is an essential framework for mapping QTL for important traits, positional cloning of genes and understanding of genome evolution.

**Results:**

We constructed a consensus linkage map of Mozambique tilapia and red tilapia using 95 individuals from two F_1_ families and 401 microsatellites including 282 EST-derived markers. In addition, we conducted comparative mapping and searched for sex-determining loci on the whole genome. These 401 microsatellites were assigned to 22 linkage groups. The map spanned 1067.6 cM with an average inter-marker distance of 3.3 cM. Comparative mapping between tilapia and stickleback, medaka, pufferfish and zebrafish revealed clear homologous relationships between chromosomes from different species. We found evidence for the fusion of two sets of two independent chromosomes forming two new chromosome pairs, leading to a reduction of 24 chromosome pairs in their ancestor to 22 pairs in tilapias. The XY sex determination locus in Mozambique tilapia was mapped on LG1, and verified in five families containing 549 individuals. The major XY sex determination locus in red tilapia was located on LG22, and verified in two families containing 275 individuals.

**Conclusions:**

A first-generation linkage map of salt tolerant tilapia was constructed using 401 microsatellites. Two separate fusions of two sets of two independent chromosomes may lead to a reduction of 24 chromosome pairs in their ancestor to 22 pairs in tilapias. The XY sex-determining loci from Mozambique tilapia and red tilapia were mapped on LG1 and LG22, respectively. This map provides a useful resource for QTL mapping for important traits and comparative genome studies. The DNA markers linked to the sex-determining loci could be used in the selection of YY males for breeding all-male populations of salt tolerant tilapia, as well as in studies on mechanisms of sex determination in fish.

## Background

Tilapia is the common name for a group of cichlid fishes native to Africa and the Middle East. The annual yield of farmed tilapia and captured tilapia reached 3.10 and 0.79 million tons, respectively, in 2009 [1], making this fish one of the most important food fishes in the world. Though tilapias normally live in freshwater, a few species of them show high salt tolerance and could be raised in brackish water or sea water [2,3]. Mozambique tilapia and its hybrids, including red tilapia are the major representatives of these euryhaline cichlids in aquaculture [4,5]. Due to the increasing lack of freshwater in the world, it would be beneficial to culture tilapia stocks in brackish or saline rearing environments to ensure a source of cheap and high-quality animal protein into the future. Based on the growth performance in salt water, Mozambique tilapia and red tilapia are competent candidates for breeding saline tilapia strains [5,6].

A genetic linkage map is an essential framework for QTL mapping of important traits, positional cloning of interesting genes and understanding of genome evolution [7-11]. The first-generation linkage maps of cultured fish species were constructed mainly by using dominant markers such as RFLP, RAPD and AFLP [8,12]. Due to the quick development of sequencing and genotyping technologies, co-dominant markers, such as microsatellites and SNPs, have replaced dominant markers for constructing linkage maps [10]. Due to their high polymorphism and application of genetic analyzers that enable high-throughput, automatic and precise genotyping, microsatellites have been the main markers used in genetic mapping of major aquaculture species such as Atlantic salmon [13], rainbow trout [14,15], channel catfish [7], grass carp [9] and Asian seabass [16]. Recently, SNPs were used in constructing a high density linkage map of salmon [17]. Several linkage maps have been constructed using microsatellite and AFLP markers [18-20] in tilapia. Two of them were constructed based on Nile tilapia and F2 hybrids of Nile tilapia and blue tilapia, respectively [18,19], and a third linkage map constructed based on a three-way cross family contained only 62 markers from Mozambique tilapia [20]. An integrated genetic linkage map is still unavailable in saline tilapia.

Sex determination is complex in vertebrates. In birds and snakes, females have a pair of ZW heteromorphic chromosomes [21], while in mammals, most males have a Y chromosome which harbors a male-determining gene SRY [22,23]. The mechanisms of sex determination are more complex in fishes, where sex is determined by different genetic and environmental factors [21]. Some closely related fishes in the same genus may have different sex-determination systems, such as XY and ZW. The DMY gene was identified as a sex-determining gene in medaka (*Oryzias latipes*), but not in other fish species [24,25]. In previous studies, markers associated with sex determination were mapped to LG1 and LG23 in Nile tilapia [26,27]. In addition, another sex determination locus was found on LG3 in the hybrid population of *O. niloticus* x *O. aureus*[19]. Nile tilapia, Mozambique tilapia and blue tilapia all are classed into the genus *Oreochromis*. The first two share a XY sex determination system while blue tilapia has a ZW one [28,29]. Though sex-determination loci in Nile tilapia and blue tilapia have attracted much attention, few reports have been published in Mozambique tilapia.

To facilitate the mapping of quantitative trait loci (QTL) for important traits and comparative genomics studies, we have constructed a consensus linkage map of Mozambique tilapia and red tilapia using microsatellites, and conducted comparative mapping. In addition, we have performed a whole genome search for sex-determining loci, and found that sex-determination loci of Mozambique tilapia and red tilapia were located on LG1 and LG22 (LG23 of a previous map [19]), respectively.

## Results

### Microsatellite makers

Sequences of 117,222 ESTs of tilapia were downloaded from Genbank, and assembled into 18121 contigs and 32034 singletons. After analyzing these unisequences using SiRoKo software [30], 1599 sequences containing microsatellites of at least seven perfect repeats were obtained, and 434 of them were further selected for the designing of primers to amplify the microsatellites in genome DNA. Among the 434 pairs of primers, 390 amplified specific products in both Mozambique and red tilapia, and 286 were informative in mapping families. In addition, 121 of the 142 markers from the previous tilapia map [19] produced informative genotypes. A total of 407 informative markers were used in the linkage analysis (Additional file 1: Table S1).

### Linkage analysis

#### Consensus linkage map

**Figure 1:**
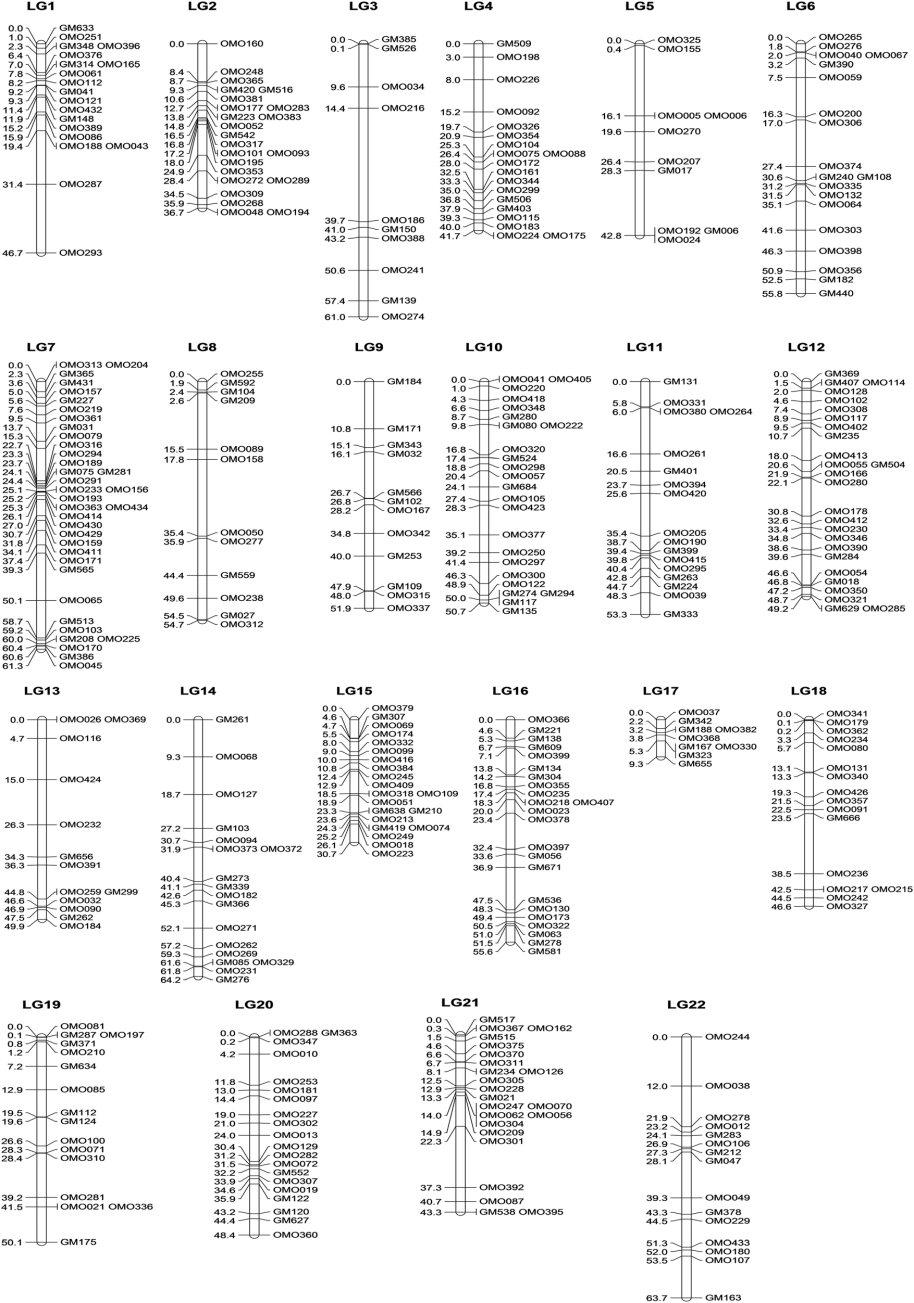
**A consensus linkage map of saline tilapia.** The location of each marker is indicated on the left side of linkage groups in Kosambi centimorgans, and names of the markers are indicated on the right side of linkage groups.

Four hundred and one markers, including 282 from ESTs and 119 from the previous tilapia map, were assigned to the consensus map, while the remaining six markers remained unmapped. The consensus map consisted of 22 linkage groups spanning 1067.6 cM (Figure 1). The number of markers per linkage group ranged from 9 to 36. The average inter-marker distance was 3.3 cM (Table 1). In most parts of the linkage map, the marker spacing was less than 20 cM, while only one marker interval on LG3 was bigger than 20 cM (Figure 1).

**Table 1:** Properties of the consensus linkage map of saline tilapia

**LG**	**Length (cM)**	**No. of loci**	**cM/marker**
1	46.7	19	2.9
2	36.7	23	2.2
3	61.0	10	6.1
4	41.7	19	2.5
5	42.8	10	6.1
6	55.8	19	3.3
7	61.3	36	2.0
8	54.7	12	4.6
9	51.9	12	4.3
10	50.7	24	2.5
11	53.3	17	3.3
12	49.2	26	2.1
13	49.9	13	4.5
14	64.2	18	4.0
15	30.7	21	1.7
16	55.6	23	2.5
17	9.3	9	1.6
18	46.6	16	3.1
19	50.1	16	3.6
20	48.4	20	2.5
21	43.3	23	2.5
22	63.7	15	4.2
Total	1067.6	401	--
Average	48.5	18.2	3.3
(±SD)	(±11.9)	(±6.2)	(±1.3)

#### Maps of Mozambique tilapia and red tilapia

**Figure 2:**
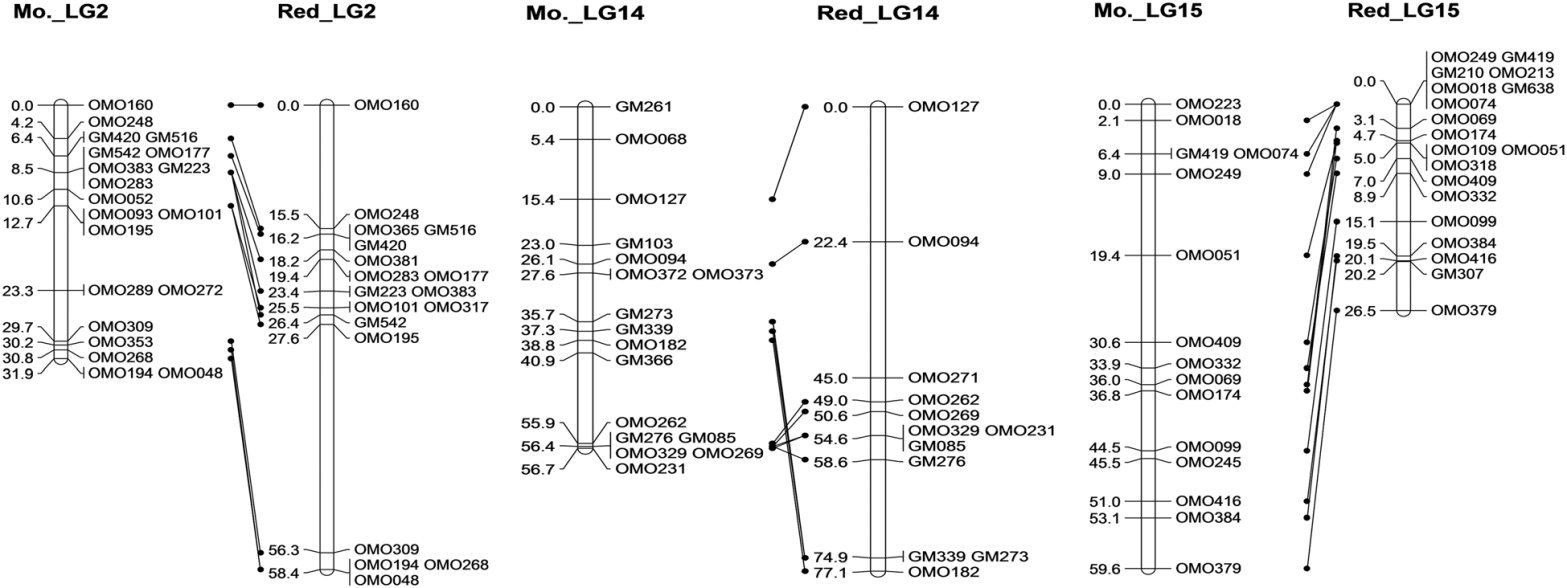
**Comparative maps of LG2, LG14 and LG15 between Mozambique tilapia and red tilapia.** The linkage groups of Mozambique tilapia are presented on the left side of each pairs of homologous linkage groups, and the linkage groups of red tilapia are presented on the right side. LG2 and LG15 show the differences of recombination rates between Mozambique tilapia and red tilapia, and LG14 shows the potential inversion.

The linkage maps of Mozambique tilapia and red tilapia were also constructed (see Additional file 2: Figure S1), respectively. These two maps contained 301 and 320 markers, and spanned 1042.5 and 984.0 cM, respectively. The number of shared markers was 232, and the total lengths of the map based on shared markers were 866.2 and 881.8 cM in Mozambique tilapia and red tilapia, respectively (see Additional file 3: Table S2). The number of linkage groups and the marker order in the two maps were basically identical, except LG14, where a region of three markers in the middle of the group in Mozambique tilapia was located at the end of the group in red tilapia. In addition, the recombination rate of LG15 in Mozambique tilapia was much higher than in the red tilapia, and that of LG2 was lower than red tilapia (Figure 2, Additional file 3: Table S2).

#### Comparison between the female and male maps

Linkage maps were also constructed for males and females (see Additional file 4: Figure S2). The maps of males and females contained 351 and 299 markers, and spanned 1104.3 and 1051.3 cM, respectively. These two maps shared 261 markers, and the total lengths of the linkage map based on shared markers were 950.8 and 1030.6 cM in males and females, respectively. The ratio of lengths of the common interval in females and males was 1.08. Though females and males had similar map lengths, the differences of recombination rates between the two genders were significant on a few linkage groups. For instance, the ratios of lengths between females and males were 0.58, 2.64 and 1.52 on LG1, LG15 and LG20, respectively (see Additional file 3: Table S2).

#### Comparison between the present map, previous maps and mapping markers onto the genome sequence of Nile tilapia

By comparing the positions of shared markers, we established the correspondence of linkage groups between the present map and the previous tilapia maps (see Additional file 5: Table S3). All 119 markers derived from 24 linkage groups of a previous map [19] were assigned to 22 linkage groups in our map. The previous groups 8, 24 and 16, 21 were merged into LG8 and LG16, respectively, reducing the linkage group numbers from 24 to 22, which corresponded to the 22 chromosomes in tilapia. For convenience, we named our linkage groups according to the previous maps with the exception of the former groups 21, 22, 23 and 24. The former groups 21 and 24 had been merged into groups 16 and 8, and the former groups 22 and 23 corresponded to the present groups 21 and 22, respectively.

After BLAST against the genome sequences of Nile tilapia in NCBI, all 401 markers were assigned to 185 scaffolds, including 84 of the 100 largest scaffolds in genome sequences (see Additional file 6: Table S4).

### Annotation of mapped ESTs

After BLAST against nt and nr databases in NCBI using 282 marker sequences derived from ESTs, 125 of them were annotated by known genes, including immune-related genes such as MHC I, chemokine receptor, interleukin-5 receptor, alpha-2-macroglobulin, lysosomal-associated transmembrane protein and ADAMTS-1 protein, and growth-related genes such as somatostatin and growth hormone receptor. Most annotations had a hit of E-value less than 10^-10^ (see Additional file 7: Table S5).

### Comparative mapping

**Figure 3:**
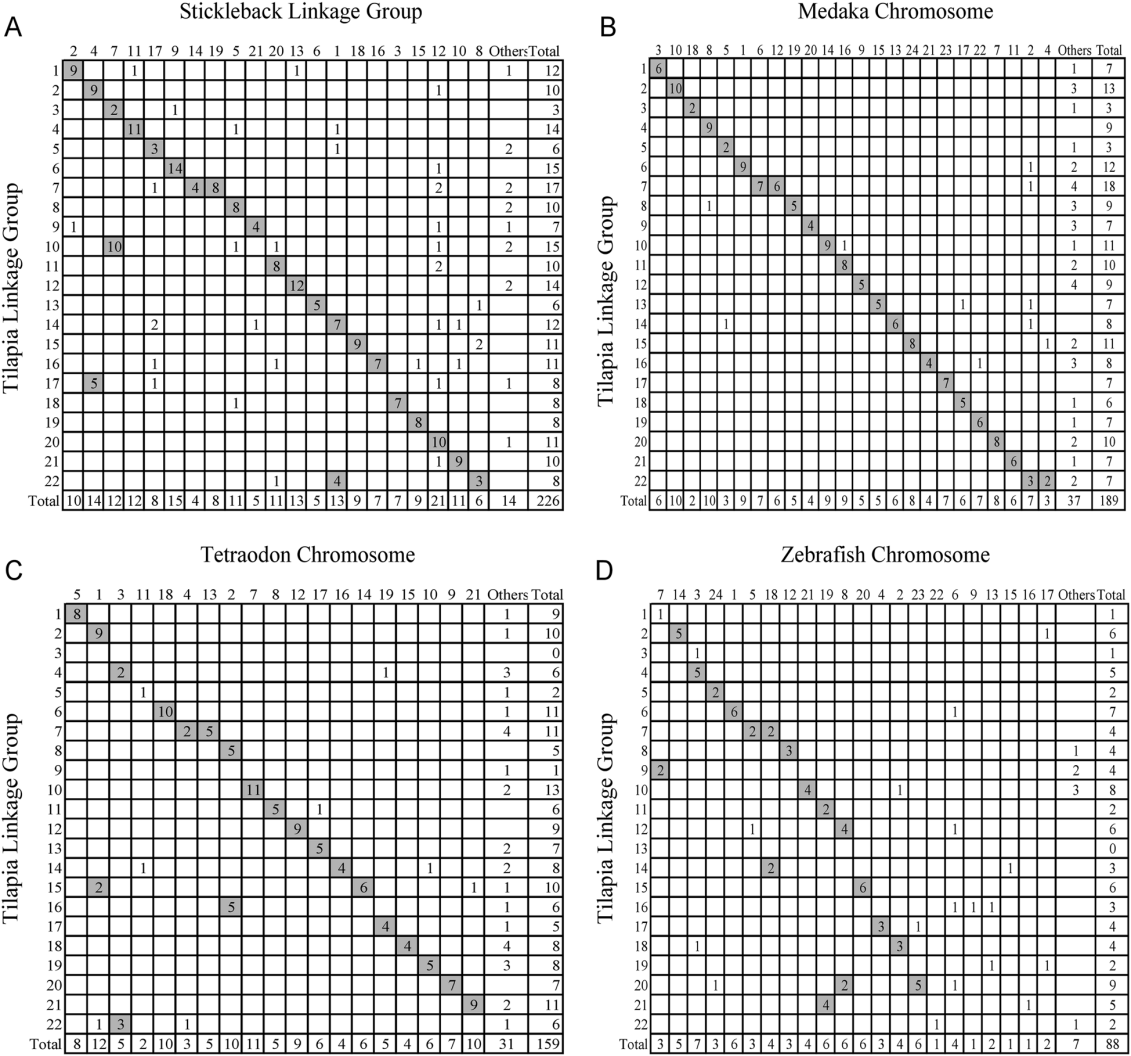
**Macrosyteny relationships between genomes of tilapia and 4 model fishes.** The number of tilapia markers with significant hits against model fishes are presented in the table, and the putative syntenic pairs are indicated by grey boxes. “Others” represents the unmapped scaffolds and contigs.

Among all 401 mapped marker sequences, 226, 188, 159, and 88 had a specific and significant hit in stickleback, medaka, pufferfish, and zebrafish, respectively. A total of 287 markers matched at least one model fish, and 188, 111, and 62 markers matched two, three, and four model fishes, respectively (Additional file 8: Table S6). Surprisingly, most of these homologous markers from the same linkage group in tilapia were located in one or two chromosomes in model fishes (Figure 3), indicating obvious homologous chromosomal relationships between tilapia and all four model fishes (see Additional file 9: Table S7).

**Figure 4:**
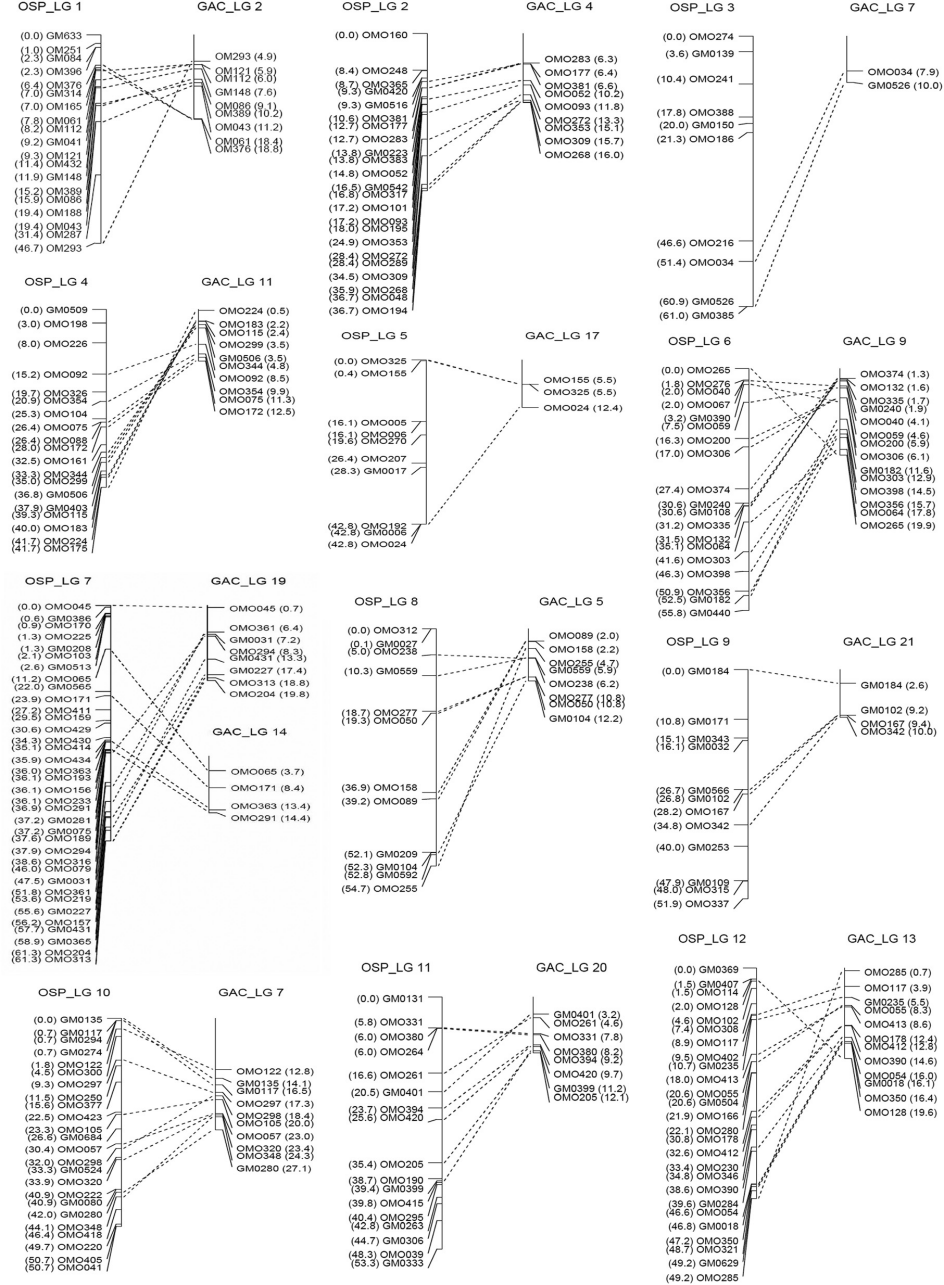
**A comparative map for LGs 1–12 between tilapia and stickleback.** The consensus linkage groups of tilapia are presented on the left side of each pairs of homologous linkage groups, and the linkage groups of stickleback are shown on the right side. The locations of markers in tilapia are indicated in Kosambi centimorgans (cM), and the locations in stickleback are indicated in physical distances (Mb).

**Figure 5:**
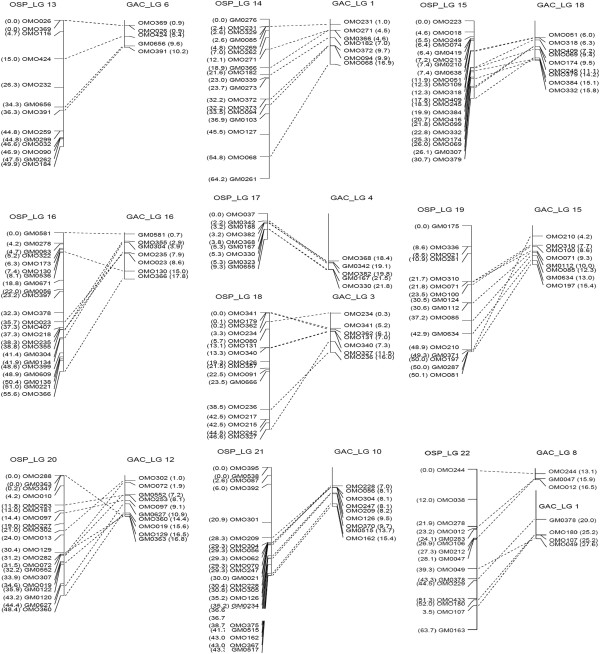
**A comparative map for LGs 13–22 between tilapia and stickleback.** The consensus linkage groups of tilapia are presented on the left side of each pairs of homologous linkage groups, and the linkage groups of stickleback are shown on the right side. The locations of markers in tilapia are indicated in Kosambi centimorgans (cM), and the locations in stickleback are indicated in physical distances (Mb).

A comparative map between tilapia and stickleback was constructed (Figures 4 and 5). A total of 212 markers were assigned to the paired linkage groups in the map. All linkage groups in tilapia also had one major homologous group in stickleback except group 7 and 22, which had two homologous groups. In addition, 14 markers of tilapia with significant hits were assigned to unmapped scaffolds in stickleback (Figure 3).

### Mapping of sex-determining loci

The nonparametric mapping identified only one sex-determining locus on LG22 in the mapping family MR-Cross 1 and another sex-determining locus on LG1 in the mapping family MR-Cross 2 (*P* < 0.005 recommended by MapQTL manual) (Tables 2, 3). Both interval mapping and MQM mapping were further used to discover potential sex determination loci, while the permutation test was used to determine the LOD value of 95% confidence interval. The results verified the results of the nonparametric mapping data, where only one sex-determining locus on LG22 was found in mapping family MR-Cross 1, and only one sex-determining locus on LG1 was found in mapping family MR-Cross 2 (see Additional file 10: Figure S3).

**Table 2:** Sex-linked markers on linkage group 22 of tilapia

**Marker**	**Position (cM)**	K*	**Degrees of freedom**	**P-value**
				
OMO244	0	8.29	1	<0.005
OMO278	21.9	10.36	1	<0.005
OMO106	26.9	15.20	1	<0.0001
GM212	27.3	15.20	1	<0.0001
GM047	28.1	13.40	1	<0.0005
OMO049	39.3	7.95	1	<0.005

* Kruskal-Wallis test statistic.

**Table 3:** Sex-linked markers on linkage group 1 of tilapia

**Marker**	**Position (cM)**	K*	**Degrees of freedom**	**P-value**
				
OMO376	6.4	21.04	3	<0.0005
GM314	7.0	21.05	3	<0.0005
OMO165	7.0	21.05	3	<0.0005
OMO061	7.8	21.05	3	<0.0005
GM041	9.2	23.62	3	<0.0001
OMO432	11.4	26.86	3	<0.0001
OMO086	15.9	32.55	1	<0.0001
OMO287	31.4	36.59	1	<0.0001
OMO293	46.7	15.79	1	<0.0001

* Kruskal-Wallis test statistic.

**Figure 6:**
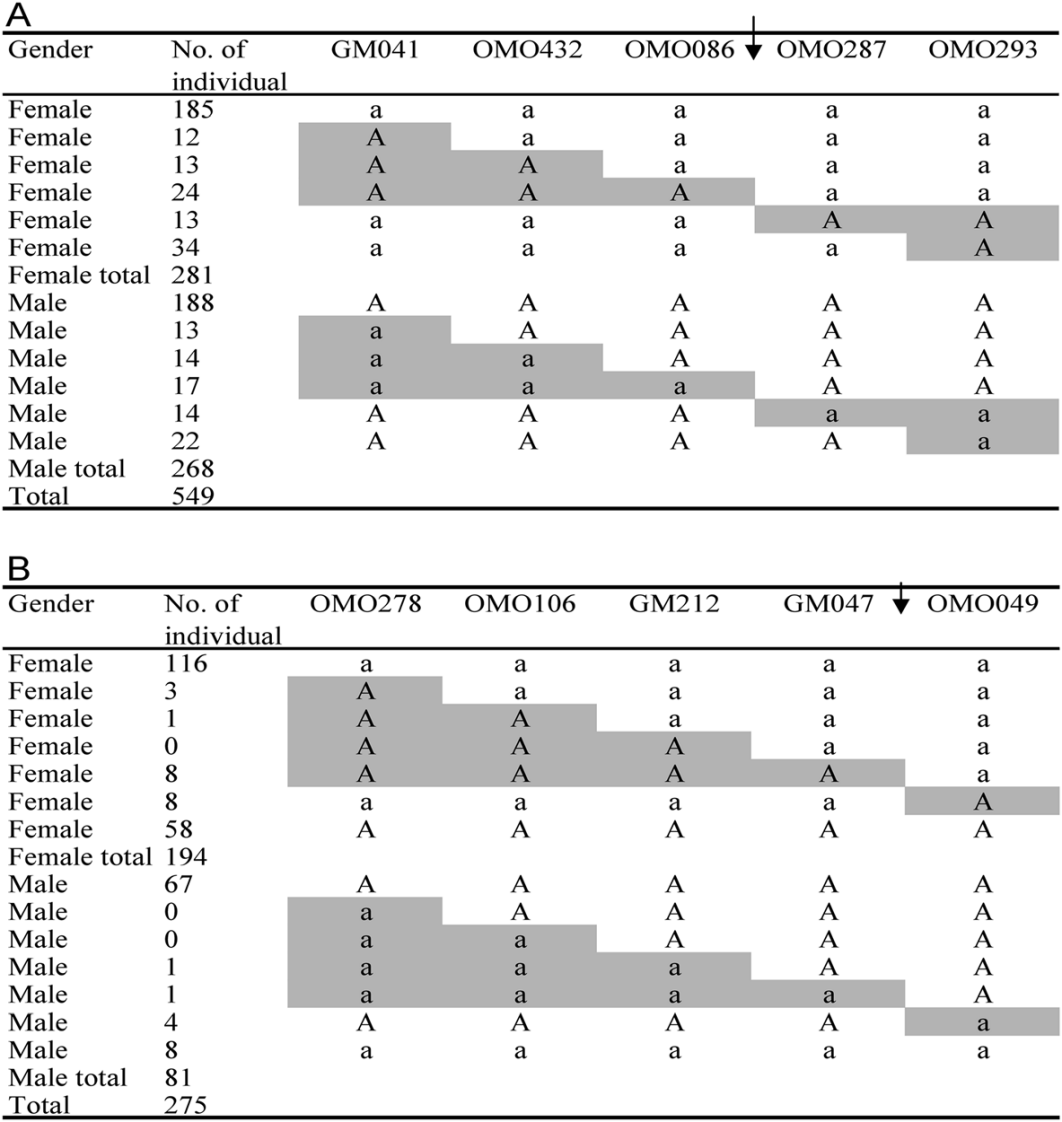
**Genotypes of DNA markers linked to the sex-determination loci on LG1 (A) and LG22 (B)**. “A” and “a” represent the alleles which come from father and mainly existed in males and females of the families, respectively. The shade indicates the recombination regions. The arrows indicate the potential positions of sex determination loci.

When we verified the sex-determining loci in seven families, all families showed significant correlations between sex and genotypes of markers from LG1 or LG22. These results indicate the locus on LG1 was the only sex-determining locus in Mozambique tilapia and hybrid tilapia produced by Mozambique tilapia males, and the locus on LG22 was the main sex-determining locus in hybrid tilapia produced by red tilapia males. The breakpoint analysis of recombination revealed that the XY sex-determining locus on LG1 was located between OMO086 and OMO287, and the XY sex-determining locus on LG22 was mapped between GM047 and OMO049 (Figure 6). However, 66 individuals (58 females and eight males, which account for approximately 30% of the progeny in the two families produced by red tilapia males) had the genotypes of opposite sex on LG22. Further genotyping was performed for LG1, and no correlation between sex and genotypes of markers from LG1 was found in these individuals.

## Discussion

### Microsatellites for linkage mapping in tilapia species

Although some microsatellites developed in one species can be used in other closely related species, the success rate of cross-species amplification is usually low [31]. In this study, microsatellites derived from the genome and EST sequences of Nile tilapia were used to construct a linkage map for saline tilapia. Among 576 microsatellites from Nile tilapia, 407 amplified specific and polymorphic products in Mozambique tilapia and red tilapia, indicating that the majority (70.7%) of microsatellites could be universally used in Nile tilapia, Mozambique and red tilapia for genetic and genomics studies.

### The linkage maps in tilapias and recombination rates

Several genetic linkage maps have been constructed in tilapias previously [18-20]. The first two maps, one in Nile tilapia, and another based on a three-way cross family, were constructed mainly using dominant AFLP markers [18,20]. Though a female map of Mozambique tilapia was constructed in the three-way cross family, it only consists of 14 linkage groups with 62 loci [20]. The latest linkage map in tilapia was constructed using an F_2_ interspecific hybrid family between Nile tilapia and blue tilapia, consisting of 24 linkage groups [19]. In the present study, we constructed the first integrated linkage map in Mozambique and red tilapia. Well-known for their high salt tolerance, Mozambique tilapia and its hybrid including red tilapia have been widely used in the aquaculture and breeding of saline tilapia [4-6]. The consensus and linkage maps of Mozambique tilapia and red tilapia, all consisted of 22 linkage groups. 282 mapped markers were derived from ESTs, and 125 of them were annotated by known genes, including genes related to immunity and growth. The potential inversion between Mozambique tilapia and red tilapia found in LG14 in the present study, along with the differences of karyotypes among tilapias reported before [32], indicates that some significant differences may exist in chromosomes between different tilapia species. With an average inter-marker distance of 3.3 cM, the present map provides a useful resource for QTL mapping of important commercial traits, comparative mapping and positional cloning of interesting genes in saline tilapia. However, we have noticed that one marker space on LG3 was still larger than 20 cM. Therefore, it is essential to map more DNA markers in this space to facilitate QTL mapping for important traits.

One hundred and nineteen microsatellite markers from 24 linkage groups of a previous map, along with 282 markers from ESTs were assigned to 22 linkage groups. The marker order on each linkage group among the present and the previous maps were almost identical (see Additional file 5: Table S3). The LG21 and LG24 of the previous map merged into LG16 and LG8, respectively. These merges reduced the number of linkage groups from 24 to 22, equal to the chromosome pair number in Mozambique tilapia and Nile tilapia [32], and resolved the discrepancy between linkage group number and chromosome number in the latest linkage map of tilapia [19].

Comparing the linkage groups between Mozambique tilapia and red tilapia revealed significant differences in the recombination rate on LG2 and LG15. Similar results have been reported in other species, such as fox and dog [33]. These results suggest that recombination rate is unequal in different genome regions and species. Different ratios of recombination rates between females and males have been reported in a number of species. Females usually have a higher recombination frequency than males. For example, the female: male recombination ratios were 8.26:1 in Atlantic salmon [34], 1.6:1 in channel catfish [7], 2.74:1 in zebrafish [35] and 2:1 in grass carp [9]. However, the linkage map of females was shorter than that of males in striped bass [36]. Nearly identical recombination rates between females and males have been referred in hybrid tilapia previously [19]. In this study, the ratio of lengths of common intervals in females and males was 1.08, indicating that males had a similar recombination frequency of the whole genome as females in tilapia.

### Syntenies between different fish species

The sequences of 226, 188, 159, and 88 of the 401 mapped markers had significant hits in the whole genome sequences of stickleback, medaka, puffer fish, and zebrafish, respectively, suggesting that stickleback is more closely related to tilapia than the other three model fishes. All linkage groups in tilapia mainly corresponded to one or two linkage groups, or chromosomes in the four model fishes (Table 3), implying the high evolutionary conservation of chromosomes in these five fish species. Some conserved syntenies of fish chromosomes also were reported in medaka [37], pufferfish [38], seabream [39], catfish [7], grass carp [9] and striped bass [36].

Among all 22 linkage groups in tilapia, LG7 corresponded to two chromosomes in all four model fishes, and LG22 corresponded to two chromosomes in stickleback and medaka, implying that these two groups may be formed by two independent fusion events. It is believed that the ancestral karyotype of cichlids consisted of 24 chromosome pairs [40]. However, how the haploid chromosome number of most tilapiines reduced to 22 remains unclear. One hypothesis is that the largest chromosome in tilapiines came from the fusion of three chromosomes [41]. However, our results suggested that the modern karyotype of tilapiines may be formed by two separate fusions of two sets of two independent chromosomes. These two fusions may have lead to the reduction of the 24 chromosome pairs to 22 pairs in most tilapiines.

Among all four model fishes compared, only medaka from superorder Acanthopterygii has a haploid chromosome number of 24, which conforms to the presumed ancestral karyotype of cichlids. In addition, each linkage group in tilapia correlates to one homologous chromosome in medaka except LG7 and LG22, each of which coincides with two chromosomes. The syntenies between proto-chromosomes in vertebrates and chromosomes in medaka have been previously speculated [37]. In the present study, the simple and clear correspondences between 22 linkage groups of tilapia and 24 chromosomes of medaka indicate that medaka may possess the most possible ancestral karyotype of cichlids in four model fishes.

A potential inversion on LG 14 was found between Mozambique tilapia and red tilapia. Differences of karyotype between Nile tilapia and Mozambique tilapia have been reported previously. Mozambique tilapia had four metacentric or submetacentric chromosomes and 40 acrocentric or subtelocentric ones, while Nile tilapia only had two metacentric or submetacentric chromosomes [32]. Our red tilapia originated from the hybrid of Mozambique tilapia and Nile tilapia. The difference of marker order on LG14 between the two tilapias may be caused by the hybrid origination of red tilapia.

### Mapping of sex determination loci

Two sex determination systems, XY and WZ have been identified in tilapias [26,28,29,42,43]. Three sex determining loci have been identified on LG1, LG3 and LG22 of tilapias [26,27], respectively, indicating the complexity of the sex determination in tilapia,. A sex-determining locus on LG3 was reported in female heterogametic (WZ-ZZ) tilapias including *T. mariae*, *O. karongae*, *O. tanganicae* and Israeli stain of *O. aureus*. A sex determining locus on LG1 was found in male heterogametic (XX-XY) Nile tilapia and *T. zillii*[26], and the sex determining locus on LG22 was only found in Nile tilapia [27]. In the present study, only one sex determination locus on LG1 was found in our reference families produced by Mozambique tilapia males, which were identified as male heterogametic. However, Cnaani et al. found that markers on both LG1 and LG3 were associated with sex in three families of Mozambique tilapia and two families of the Egyptian strain of blue tilapia, and the sex determination of these reference families could not be defined as male or female heterogametic [26]. Mozambique tilapia and blue tilapia were known as male heterogametic and female heterogametic, respectively [28,29]. Our results are identical to the traditional view and differ from the results of Cnaani et al. [26]. This divergence may be caused by the different genetic backgrounds of reference families and strains. Interspecies crosses were prevalent in the tilapias, and most of the hybrids were fertile and could reproduce offspring as purebred fish [44,45]. These hybrids may spread in the farmed strains as well as in wild populations, and lead to the complex pattern of sex determination in some tilapia strains.

Since the sex-determining locus on LG1 was identified mainly in tilapias with the XY sex determination system, and the sex-determining locus on LG3 was identified mainly in tilapias with the WZ system [26], we may conclude that the sex determining locus on LG1 determined the male heterogamete and the sex determining locus on LG3 determined the female heterogamete in tilapiine species. As these sex determining loci existed in closed species from the genus of both *Oreochromis* and *Tilapia*[26], it seems that the two sex-determining loci may both emerge prior to the differentiation of *Oreochromis* and *Tilapia*, and underwent independent evolution in different species. Alternatively, the tilapiine species may only have one ancestral sex determination locus, which more likely to be the sex-determining locus on LG3 as predicted by some researchers [26]. After the differentiation of *Oreochromis* and *Tilapia*, another sex-determining locus appeared and spread to specific species by interspecific hybridization.

We have also identified a XY sex-determining locus on LG22 in red tilapia. This sex-determining locus was reported only in Nile tilapia [27]. Our local red tilapia strain in Malaysia and Singapore originating from the hybrid between Nile tilapia and Mozambique tilapia [4]. The sex-determining locus on LG22 in red tilapia may originate from Nile tilapia instead of Mozambique tilapia. As it only was found in Nile tilapia and red tilapia, the sex-determining locus on LG22 may have a later origination than the sex-determining loci on LG1 and LG3. In this study, the sex-determining loci on LG1 and LG22 were identified in different types of families, which were produced by Mozambique tilapia males or red tilapia males, respectively. There was no family found to have these two sex-determining loci at the same time. Further research is needed to understand the interactions between the sex-determining loci on LG1 and LG22.

About 30% of individuals from two families produced by the red tilapia males showed no association between sex and genotypes of LG1 or LG22, indicating that there are more genetic or environment factors which may be involved in the sex determination in red tilapia. Due to its hybrid origination, red tilapia may have more complex mechanisms of sex determination than Mozambique tilapia.

## Conclusions

We constructed a first consensus linkage map of Mozambique tilapia and red tilapia. The map consisted of 22 linkage groups, spanning 1067.6 cM and containing 401 microsatellite markers derived mainly from ESTs. Comparative mapping between tilapia and four model fishes indicates that high evolutionary conservation of chromosomes existed in these fish species. Two separate fusions of two sets of two independent chromosomes may lead to a reduction of 24 chromosome pairs in their ancestor to 22 pairs in tilapias. Sex-determining loci in Mozambique tilapia and red tilapia were mapped on LG1 and LG22, respectively. Our linkage map and markers linked to the sex-determining loci provide a useful resource for genetic improvement of salt tolerance tilapia and future genomics study in fish.

## Methods

### Mapping families and DNA isolation

Two mapping families, MR-Cross 1 and MR-Cross 2, were established by crossing Mozambique tilapia and red tilapia. MR-Cross 1’s parents were a Mozambique tilapia female and a red tilapia male, and MR-Cross 2’s parents were a Mozambique tilapia male and a red tilapia female. The Mozambique tilapias were F1 offspring of wild population coming from South Africa, and the red tilapias were from a local red tilapia strain in Malaysia and Singapore, which originated from a hybrid between Mozambique tilapia and Nile tilapia. Fish were raised in the marine fish facility of Temasek Life Sciences Laboratory. The mapping population consisted of 470 individuals, 142 from MR-Cross 1 and 328 from MR-Cross 2. All markers were genotyped in 95 individuals, 47 from MR-Cross 1 and 48 from MR-Cross 2. The markers associated with sex were further genotyped in the remaining 375 individuals.

For verifying the sex determination loci in tilapia, additional five families containing 354 individuals have been established. These reference families included four Mozambique tilapia families and one hybrid family (Mozambique tilapia♀x red tilapia♂). Fin clips were sampled from each parent and offspring, and stored in 75% ethanol for subsequent DNA extraction with the method described by Yue and Orban [46].

### Markers and primers

ESTs of tilapia were downloaded from NCBI database, and assembled using SeqMan NGen 2.0 software [47] with default setting. SiRoKo software [30] was used to screen the unisequences containing microsatellites. Primers were designed and used to amplify these microsatellite sequences in the genomic DNA of 3 unrelated Mozambique tilapias. The primers which amplified specific productions in all 3 fishes were further labeled at 5^′^ end of the forward primer with either a Fam or Hex fluorescent dye. For comparison with the previous linkage map of *Oreochromis spp*[19], 142 markers were selected from it, and their primers were synthesized and tested in Mozambique tilapias as described above. All labeled primers were used to genotype the parents, two Mozambique tilapias and two red tilapias, and the informative primers were further used in the genotyping of mapping families (Additional file 1: Table S1).

### Genotyping

PCR amplification was carried out for each sample in a 25 μL volume containing 10 ng genomic DNA, 1 × PCR buffer, 100 μM of each dNTPs, 0.2 μM forward and reverse primers, 1 unit Taq DNA polymerase (FINNZYMES, Espoo, Finland). The reactions were performed in thermal cycler (MJ Research, CA, USA) using the following profile: one cycle of 3 min at 94°C, 38 cycles of 30 sec at 94°C, 30 sec at 50°C , 55°C or 60°C and 45 sec at 72°C , followed by a prolonged extension of 5 min at 72°C . PCR products were resolved on an ABI3730xl Genetic Analyzer (Applied Biosystems, CA, USA) and genotyped against the internal size standard of GeneScan-500 ROX using software GeneMapper 4.1 (Applied Biosystems, CA, USA).

### Map construction

JoinMap 4.0 software was used for linkage analysis and map construction [7]. The Kosambi mapping function was applied in the analysis. The grouping of makers was performed with a LOD threshold of 4.0. When the map was calculated, “ripple” was performed after adding each marker. The best-fitting position of each marker was examined based on the goodness-of-fit test (chi-square). Three-round mapping was performed for each linkage group. At first, grouping of markers was performed for mapping families MR-Cross 1 and MR-Cross 2, respectively, then the homologue linkage groups from each family were combined, and the consensus map was constructed.

For comparing maps of different tilapias and sexes, maternal and paternal population nodes were created from the dataset of each family, respectively. Grouping of was performed for the population nodes of each parent of the mapping families. The homologous groups from the same gender or same strain were combined, and the consensus maps of Mozambique tilapia, red tilapia, female and male were calculated, respectively.

### Annotation of mapped unisequences

All mapped unisequences were used to do BLAST against nt and nr databases in NCBI. The cutoff E-values were e < 10^-5^ for BLASTX and BLASTN. The best hits were regarded as the annotations of unisequences.

### Comparative mapping

The whole genome sequences of stickleback, medaka, pufferfish and zebrafish were downloaded from Ensembl (http://www.ensembl.org/info/data/ftp/index.html). These sequences were formatted as databases in a local computer, and the DNA sequences of the mapped markers were used to do similarity searches against the databases by using BLAST software. Hits with e < 10^-5^ were considered as significant. In cases where the search of one sequence hits two or more loci with less than 100 fold difference of the E-value, the sequence was considered to be duplicated in the genome, and the marker located in it wasn’t used in the further comparative analysis. The comparative map was drawn using MapDisto software (ver. 1.7) [48].

### Mapping of sex-determining loci

The sex of each individual was identified by the appearance of gonopore combined with dissection. Mapping of sex-determining loci was performed using the nonparametric mapping (Kruskal-Wallis analysis), interval mapping and MQM mapping in MapQTL 4.0. According to the recommendation of the manual for MapQTL software, the statistical significant level in nonparametric mapping was set as *P* < 0.005. Permutation Test was used to determine the significance threshold of the LOD score for interval mapping and MQM mapping.

A total of 824 individuals from seven families were genotyped to verify the sex-determining loci in different families. Five markers from LG1, including GM041, OMO432, OMO086, OMO287 and OMO293, were used to genotype 549 individuals from 4 Mozambique tilapia families and one hybrid family (red tilapia♀x Mozambique tilapia♂), and OMO278, OMO106, GM212, GM047 and OMO49 from LG22 were genotyped in 275 individuals from two hybrid families (Mozambique tilapia♀x red tilapia♂).

## Competing interests

The authors declare that they have no competing interests.

## Authors’ contributions

GHY initiated and supervised the study. FL, FS, JL, JHX, GL and RJT carried out the experimental work. FL performed the data analysis and drafted the manuscript. GHY finalized the manuscript. All authors read and approved the final manuscript.

## Supplementary Material

Additional file 1 Table S1 Primer sequences of mapped microsatellite markers in saline tilapia. Click here for file

Additional file 2 Figure S1 A comparative map between Mozambique tilapia and red tilapia. Click here for file

Additional file 3 Table S2 Recombination ratios between Mozambique tilapia and red tilapia, and female tilapia and male tilapia. Click here for file

Additional file 4 Figure S2 Linkage maps of male and female in tilapia. Click here for file

Additional file 5 Table S3 Syntenies between the current and previous linkage maps in tilapia. Click here for file

Additional file 6 Table S4 Locations of mapped markers in whole genome sequences of Nile tilapia. Click here for file

Additional file 7 Table S5 Annotations of mapped microsatellites derived from ESTs in tilapia. Click here for file

Additional file 8 Table S6 Putative othologous loci between the genomes of tilapia and four model fish species. Click here for file

Additional file 9 Table S7 Syntenies between different fish species. Click here for file

Additional file 10 Figure S3 Mapping of sex determination loci in LG1 (A) and LG22 (B). Click here for file
