# Supplementary material for: A microsatellite-based linkage map of salt tolerant tilapia (Oreochromis mossambicus x Oreochromis spp.) and mapping of sex-determining loci
Source: BMC Genomics. 2013 Jan 28;14:58. doi: 10.1186/1471-2164-14-58 (PMC3565888; doi:10.1186/1471-2164-14-58)
Supplement: Additional file 2 Figure S1 — A comparative map between Mozambique tilapia and red tilapia. [file 1471-2164-14-58-S2.pdf]

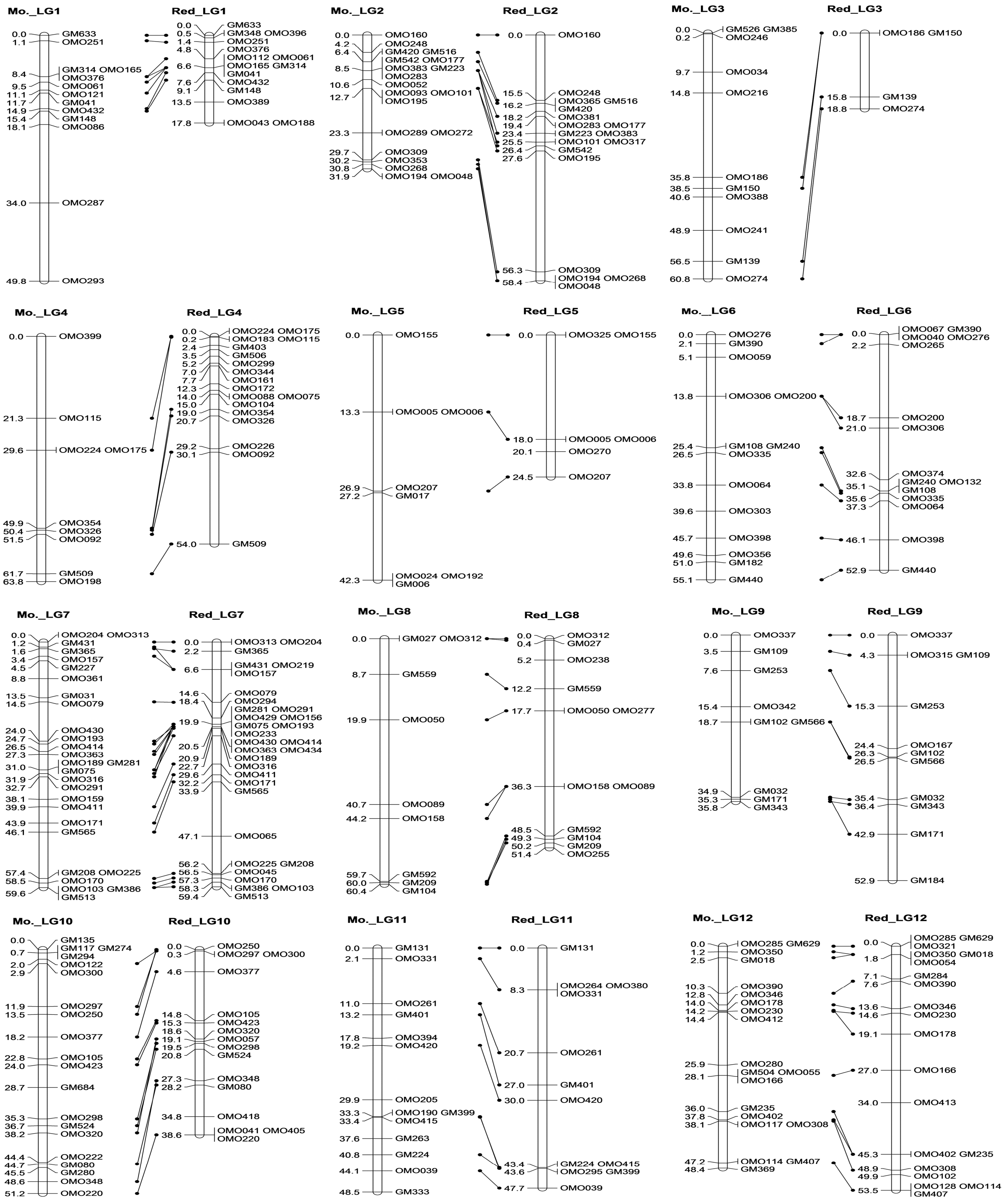

Figure S1. Comparative map between Mozambique tilapia and red tilapia (LG1-12)

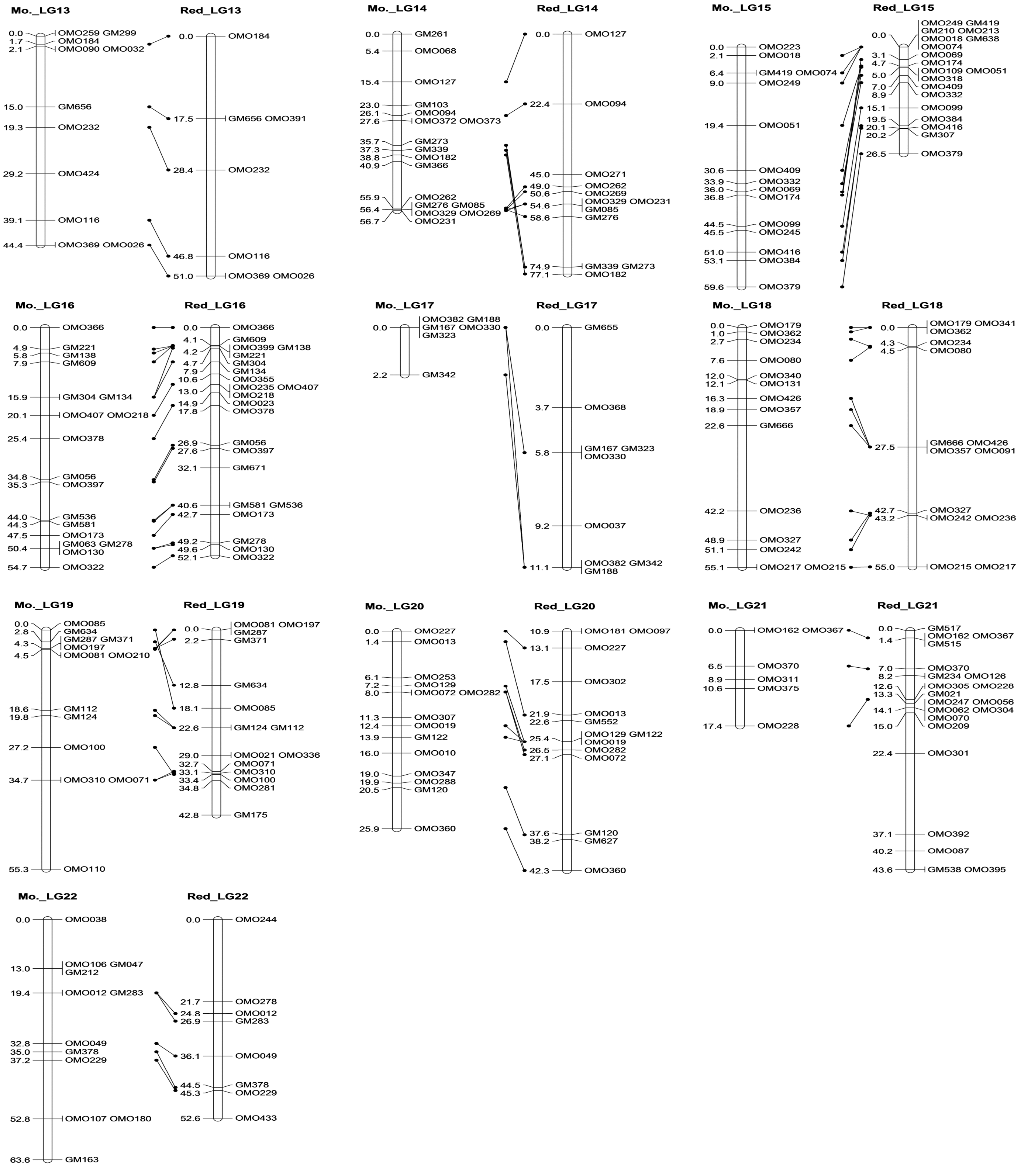

Figure S1. Comparative map between Mozambique tilapia and red tilapia (LG13-22)
