# Supplementary figures and images for: A microsatellite-based linkage map of salt tolerant tilapia (Oreochromis mossambicus x Oreochromis spp.) and mapping of sex-determining loci
Source: BMC Genomics. 2013 Jan 28;14:58. doi: 10.1186/1471-2164-14-58 (PMC3565888; doi:10.1186/1471-2164-14-58)

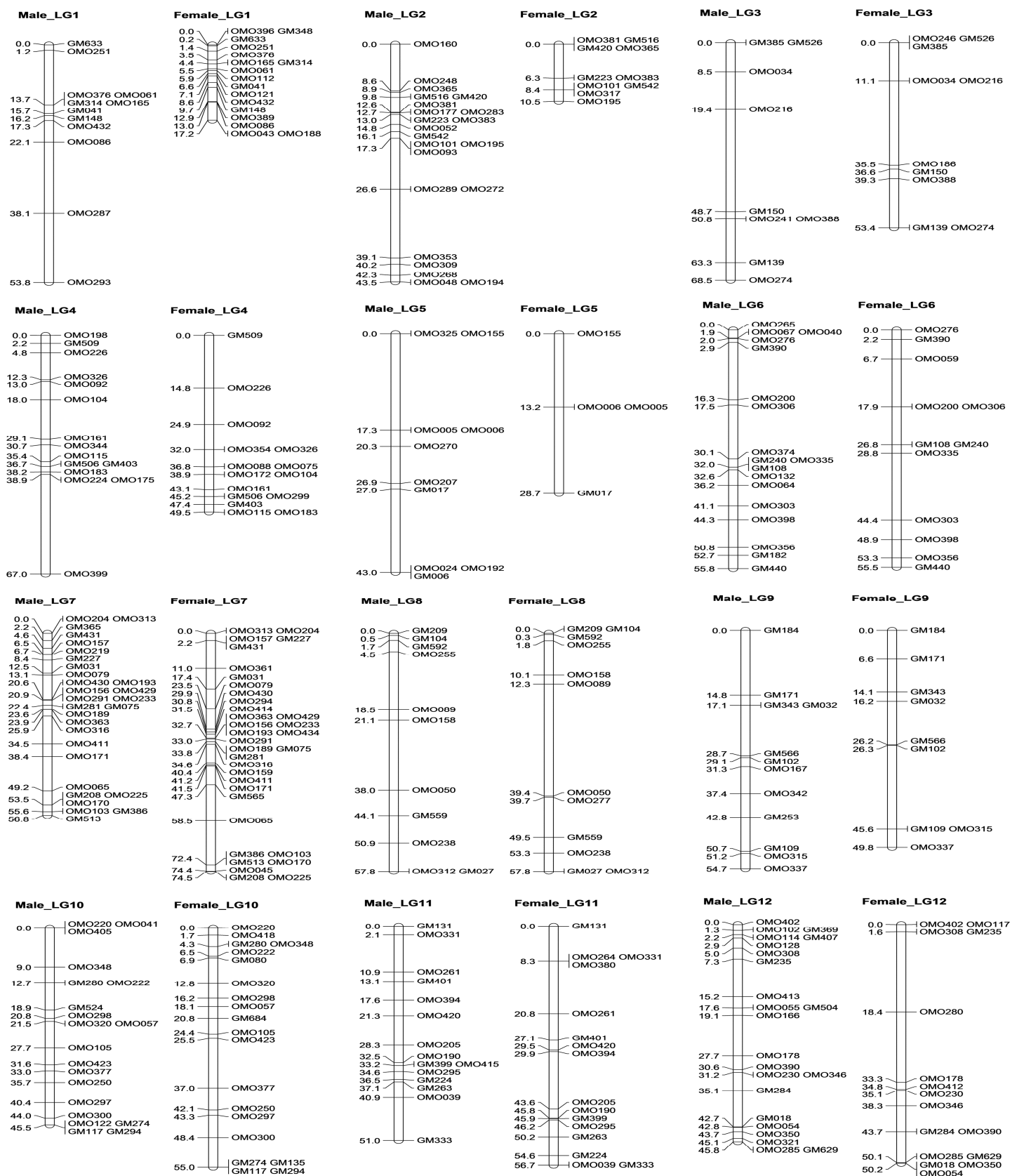

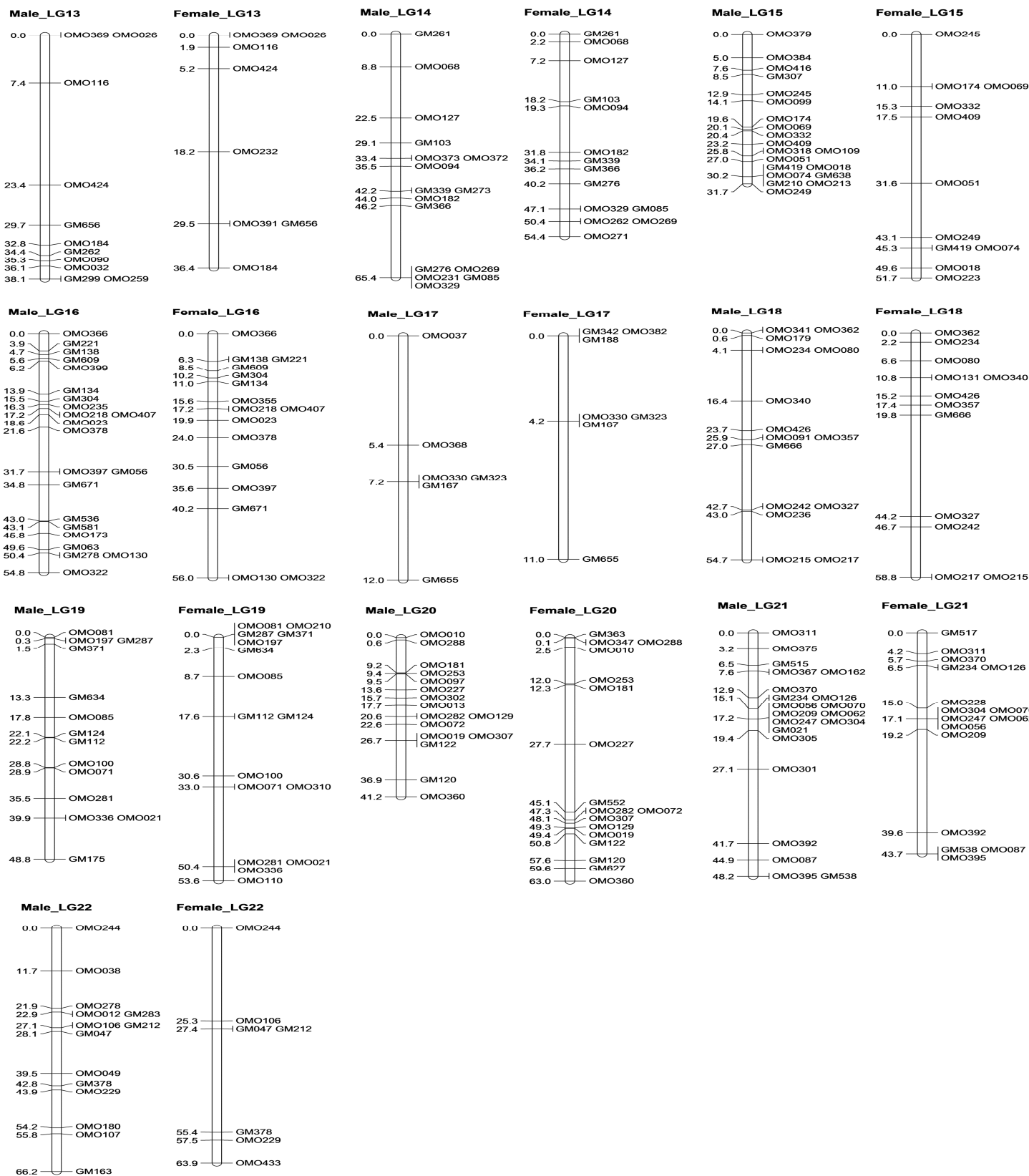

Figure S2. Linkage maps of male and female (2)

Supplement: Additional file 4 Figure S2 — Linkage maps of male and female in tilapia. [file 1471-2164-14-58-S4.pdf]
