# Supplementary material for: A microsatellite-based linkage map of salt tolerant tilapia (Oreochromis mossambicus x Oreochromis spp.) and mapping of sex-determining loci
Source: BMC Genomics. 2013 Jan 28;14:58. doi: 10.1186/1471-2164-14-58 (PMC3565888; doi:10.1186/1471-2164-14-58)
Supplement: Additional file 10 Figure S3 — Mapping of sex determination loci in LG1 (A) and LG22 (B). [file 1471-2164-14-58-S10.pdf]

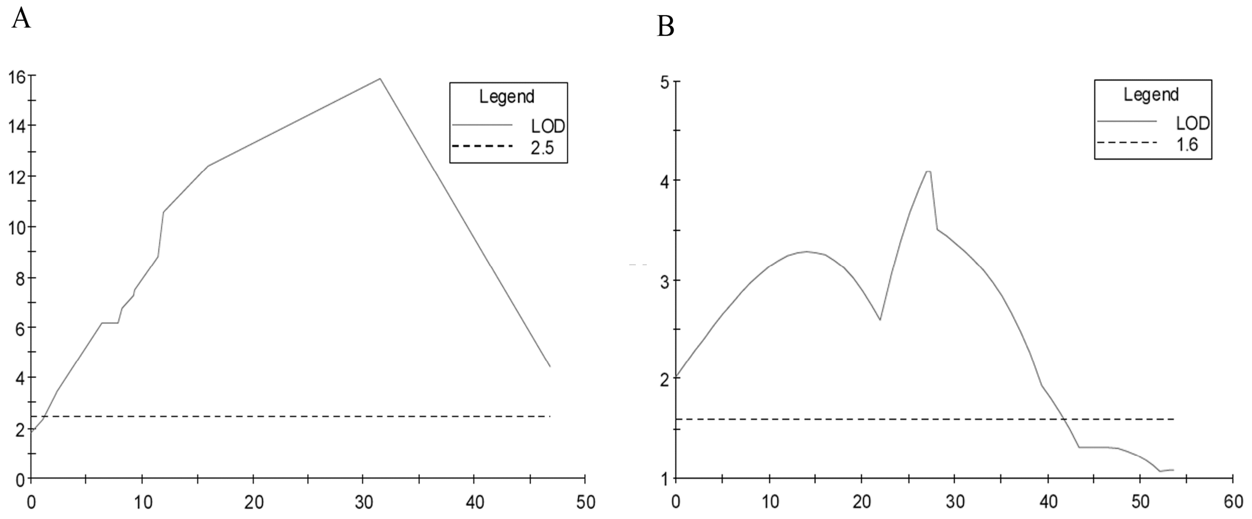

Figure S3. Mapping of sex determination loci in LG1(A) and LG22(B)
The dotted lines indicate the significance thresholds of the LOD score in linkage groups.
